# Transient Thermal Analysis Model of Damaged Bearing Considering Thermo-Solid Coupling Effect

**DOI:** 10.3390/s22218171

**Published:** 2022-10-25

**Authors:** Yali Sun, Chong Zhang, Xing Zhao, Xiaodong Liu, Chang Lu, Jiyou Fei

**Affiliations:** 1 College of Mechanical Engineering, Dalian Jiaotong University; Dalian 116028, China; 2 College of Locomotive and Rolling, Dalian Jiaotong University; Dalian 116028, China; 3 PLA Army Academy of Artillery and Air Defense, Shenyang 110000, China

**Keywords:** thermal-solid coupling, bearing fault diagnosis, heat generation and transfer, transient thermal model

## Abstract

As one of the important parameters of bearing operation, temperature is a key metric to diagnose the state of service of a bearing. However, there are still some shortcomings in the study of the temperature variation law for damaged bearings. In this paper, according to the structural characteristics of bearings, the influence law of thermal-solid coupling effect on bearing structure is considered, and a novel transient temperature analysis model of damaged bearings is established. First, a quasi-static analysis of the bearing is performed to obtain the variation laws of the key parameters of the bearing under thermal expansion. Then, the load variation law of the bearing under the condition of damage is discussed, and the heat generation and heat transfer of the damaged bearing during operation are studied. Based on the thermal grid method, a transient temperature analysis model of the damaged bearing is developed. Finally, the model is tested experimentally and the influence of the rotate speed and load on the bearing temperature variation is analyzed. The results show that the established model can effectively predict the temperature variation and thermal equilibrium temperature of damaged bearings.

## 1. Introduction

The rolling bearing is one of the most important components of high-speed trains, and plays a key role in in safety of transportation. However, the bearing easily produces defects, due to its hostile working environment [1]. Therefore, the detection of the state of bearings is quite important. However, the existing non-contact detection methods often ignore the influence of temperature on the important parameters of faulty bearings [2] (such as characteristic frequency). What should be noticed is that the faulty bearing is a system affected by the thermal-solid coupling effect. The structure size, contact load and deformation between components and lubricating oil characteristics will affect the heat generation and transfer of the bearing, and the temperature will promote the change of the features of oil, the size and performance of component structure and other parameters that affect bearing vibration characteristics. The bearing will achieve thermal equilibrium in the constant interaction and change. The contact load and deformation of the faulty bearings are quite complex, which makes the thermal analysis of such a system difficult, and leads to inaccurate basis of bearing detection and evaluation. Therefore, it is of great significance to understand the thermo-solid coupling effect of faulty bearings in operation and establish an accurate temperature prediction model for faulty rolling bearings.

Currently, research on bearing thermal analysis focuses on lubrication, heat generation and heat transfer. The bearings must be lubricated during operation, and the most important function of the lubricating oil is to provide a film of lubrication between the parts of the bearings. The thickness of the lubricant oil film depends on the surface roughness [3], structure [4], load [5], speed [6] and temperature [7] of the bearing. Dowson and Higginson [8] proposed the classical oil film thickness calculation formula, which can calculate the oil film thickness under constant temperature based on elastic mechanics without considering the influence of temperature changes, and the calculated oil film thickness is slightly higher than the actual state [9]. Since then, several scholars have modified and refined this classical formula. For example, Forster et al. [10] and Echavarri Otero et al. [11] newly calculated the oil film thickness by adding empirical coefficients, and some scholars considered the non-Newtonian shear thinning effect when calculating the oil film thickness, such as Liu et al. [12] and Shirzadegan et al. [13]. In addition, some scholars have considered the influence of thermal effect, such as Wang et al. [14] and Echa-Varri Otero et al. [15].

The heat generated by the roll bearing comes mainly from friction heat production between the roll body and the racetrack, the roll body and the cage, and the inner loop guide surface and the cage. From this point of view, the heat generated by a bearing is mainly frictional, and therefore it will be affected by factors such as the bearing velocity, load and structural parameters. For the calculation method of heat generated, Palmgren [16] took the lead in proposing the global method of calculation, and obtained the empirical formula of bearing friction torque by experimental method, and multiplied the friction torque by the bearing speed to obtain the global heat production of the bearing. Since then, several scholars have developed improvements based on the global approach. For example, Stein and Tu [17] further analyzed the influence of preloading force based on the global method; Kim et al. [18] considered the influence of bearing load and speed on friction torque; in addition, other scholars have made improvements [19]. However, the global approach still has limitations, and the specific heating position of the bearings cannot be obtained in this way. Furthermore, some scholars proposed the local method to calculate the bearing temperature [20]. This method calculates the heat production at each contact position according to the mechanical and kinematic relations of the bearing, which can clarify the heat production and heat transfer rules of each part of the bearing, and the results are more accurate [21,22].

The heat distribution of rolling bearings mainly refers to temperature transfer, such as heat transfer and heat convection in bearings.

Bearing heat distribution mainly refers to temperature transfer, primarily heat transfer and heat convection. The mode of heat transfer and the final distribution of temperature will alter the lifetime of the bearing. The calculation methods for heat transfer and temperature distribution of bearings are mainly divided into finite element method [23] and thermal network method [24]. Compared with the finite element method, the thermal network method has advantages such as mesh density, settable nodes and easy setting of boundary conditions [25]. Some scholars have studied the number of nodes [26], the distribution form [27] and the boundary setting method [28] of the network, and further developed the transient thermal network model of the bearing system [29], which can forecast the bearing temperature more precisely than the original steady state model. 

In summary, there have been some advances and achievements in existing thermal analysis models for bearings, but how to accurately judge the temperature rise and thermal equilibrium of bearings under damage conditions remains a difficult problem for bearing detection. In particular, most scholars disregard the variations of heat-affected bearing components in the construction of thermal analysis models for bearing, so that there is still some bias in the calculation process. Moreover, the effect of bearing damage on the temperature law is not taken into account. Nonetheless, in the actual operation of a bearing, there is an interplay between the bearing structure, lubrication properties, load, working conditions and other key factors, as well as the bearing temperature, under which the thermal equilibrium of the bearing is reached progressively. However, many factors drastically change after bearing damage, making the process of temperature rise and thermal balance of damaged bearings extremely complicated. Therefore, it is important to model the thermal analysis of the bearing by taking into account the thermal-solid coupling effects of the damage.

In this paper, an analytical model of temperature rises and thermal equilibrium temperature of injured bearings considering the influence of thermal-solid coupling effect is recommended. The interaction among structural parameters, contact load, heat generation, working conditions and the temperature of faulty rolling bearing was analyzed, and the predicting accuracy of the model was tested by verification experiments. This paper is structured as follows: the first section introduces the research status of thermal analysis model of rolling bearings in high-speed trains, and makes clear the difference of heat generation by contact load between good and faulty bearings. Section 2 describes the relationship between the structure parameters and force and motion of the roller, and proposes the load distribution calculation method of the faulty rolling bearing. Section 3 analyzes the calculation method of heat generation and transfer of faulty bearings during operation. Section 4 proposed the method of node division in thermal network and the calculation flow of transient temperature of the faulty rolling bearing. In Section 5, the corresponding verification experiments are carried out to prove the correctness of the temperature prediction model of the faulty bearing, and the influence of rotate speed and radial load on the temperature rise process is analyzed.

## 2. Dynamic Analysis of Damaged Bearings under the Influence of Thermal Expansion

### 2.1. Analysis of Bearing Motion and Dynamics

The accurate analysis of bearing mechanical characteristics is the premise of establishing a bearing thermal analysis model. The contact load, motion parameters and contact deformation among the internal components of high-speed rolling bearings have an important influence on the friction heat production in the process of operation. The accuracy of the calculation of these parameters will directly affect the accuracy of the thermal analysis model of bearings. Therefore, before establishing the bearing thermal analysis model, it is necessary to conduct accurate motion and force analysis on the rolling bearing first.

**Figure 1:**
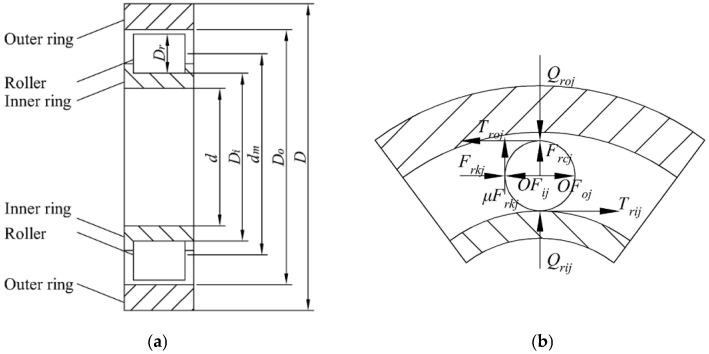
Schematic diagram of roller bearing: (**a**) schematic diagram of bearing structure; (**b**) the forces of a roller.

The schematic diagram of cylindrical rolling bearing is shown in Figure 1a. Assuming that the outer ring is fixed and there is no sliding phenomenon of the roller, the force analysis of a single roller is shown in Figure 1b. The rotational speed of the inner ring and roller are *ω_i_* and *ω_r_*. *μ_rij_*, *μ_roj_* and *μ_rkj_* are friction coefficients between the *j*th roller and the inner ring, the outer ring and the cage, respectively. *Q_rij_* and *Q_roj_* are the contact loads between roller and inner ring and outer ring, respectively. *F_rkj_* is the normal force between the roller and the cage, *T_rij_* and *T_roj_* are the friction force of the roller, *OF_ij_* and *OF_oj_* are the dynamic pressure of the roller from the lubricating oil.

According to the motion relationship shown in the figure, the relative sliding velocities (Δ*V_rij_*, Δ*V_roj_*) and average velocities (*V_rij_*, *V_roj_*) of the *j*th roller with the inner and outer rings can be obtained, which can be expressed by the following formula:

(1) $$\left\{\begin{array}{l} \Delta V_{rij}=\frac{1}{2}d_{m}\left[\left(1-\frac{D_{r}}{d_{m}}\right)\omega_{i}-\frac{D_{r}}{d_{m}}\omega_{r}\right]\\ \Delta V_{roj}=-\frac{1}{2}D_{r}\omega_{r} \end{array}\right.$$

(2) $$\left\{\begin{array}{l} V_{rij}=\frac{1}{4}d_{m}\left[\left(1-\frac{D_{r}}{d_{m}}\right)\omega_{i}+\frac{D_{r}}{d_{m}}\omega_{r}\right]\\ V_{roj}=\frac{1}{4}D_{r}\omega_{r} \end{array}\right.$$

where *d_m_* is the pitch diameter of the bearing and the *D_r_* is the diameter of the roller.

Further, the force balance equations for the bearing under stable operation can be obtained, which can be expressed in the following formulae, respectively:

(3) $$\left\{\begin{array}{l} OF_{ij}+T_{rij}-\left(OF_{oj}+T_{roj}\right)\pm F_{rkj}=0\\ Q_{rij}+F_{rcj}-Q_{roj}\pm \mu_{rkj}F_{rkj}=0\\ Q_{r}-\sum_{j=1}^{Nr}Q_{ioj}\cos\theta_{j}=0\\ \sum_{j=1}^{Nr}F_{rkj}=0\\ T_{rij}+T_{roj}-\mu_{rkj}F_{rkj}=0 \end{array}\right.$$

where *Q_r_* is the radial load of the rolling bearing, *θ_j_* is the angular position of the roller, Nr is the number of the rollers, and *F_rcj_* is the centrifugal force of the roller, which can be expressed by the following formula:

(4) $$F_{rcj}=\frac{1}{2}m_{r}d_{m}\omega_{r}^{2}$$

where *m_r_* is the quality of the roller.

By taking into account the thermal effect and shearing-thinning, the contact loads between roller and the inner and outer ring can be solved by the following formula:

(5) $$\left\{\begin{array}{l} Q_{rij}=K_{rij}{\left(\delta_{rij}+0.13h_{rij}\right)}^{10/9}\\ Q_{roj}=K_{roj}{\left(\delta_{roj}+0.13h_{roj}\right)}^{10/9} \end{array}\right.$$

where *K_rij_* and *K_roj_* are the stiffness coefficients of the inner and outer rings, respectively. *δ_rij_* and *δ_roj_* are the deformation degrees of contact. *h_rij_* and *h_roj_* are the minimum oil film thickness between the roller and the inner and outer ring, which can be obtained by the following formula:

(6) $$\left\{\begin{array}{l} h_{rij}=2.65\frac{\varphi_{t}}{\varphi_{nc}}\left(\frac{\alpha_{p}^{0.54}{\left(\eta_{0}\Delta V_{rij}\right)}^{0.7}R_{rij}^{0.43}L_{r}^{0.13}}{E^{0.03}Q_{rij}^{0.13}}\right)\\ h_{roj}=2.65\frac{\varphi_{t}}{\varphi_{nc}}\left(\frac{\alpha_{p}^{0.54}{\left(\eta_{0}\Delta V_{roj}\right)}^{0.7}R_{roj}^{0.43}L_{r}^{0.13}}{E^{0.03}Q_{roj}^{0.13}}\right) \end{array}\right.$$

where *η*_0_ and *α_p_* are the dynamic viscosity and viscosity-pressure coefficients of oil, which are affected by the temperature. *R_rij_* and *R_roj_* are the equivalent curvature radius of the contact between roller and inner and outer rings, *L_r_* is the length of roller, *φ_t_* is the oil film thermal correction coefficient and *φ_nc_* is the non-Newtonian fluid correction coefficient, which can be obtained according to the literature [30].

The total deformation of the rolling bearing can be expressed by the following formula:

(7) $$\delta_{rj}=\left(\delta_{\max}+0.5H_{ol}-\left(h_{ri1}+h_{ro1}\right)\right)\cos\theta_{j}+\left(h_{rij}+h_{roj}-0.5H_{ol}\right)$$

where *H_ol_* is the bearing clearance.

The bearing clearance *H_ol_* in the thermal expansion state can be calculated as follows.

(8) $$H_{ol}=H_{0}+\Delta_{r}-\left(\Delta D_{i}+\Delta D_{o}+2\Delta D_{r}\right)$$

where *H_o_* is the initial clearance of the bearing, Δ*_r_* is the increment of the clearance after thermal expansion, and Δ*D_i_*, Δ*D_o_* and Δ*D_r_* are the diameter changes of the rollers, inner and outer rings, caused by the change of the bearing temperature, which can be solved by the following formula:

(9) $$\left\{ \begin{array}{c} \Delta D_{i}=\frac{2\left(I_{i}+\Delta I_{i}\right)\left(\frac{D_{i}}{d}\right)}{\left[\left[\frac{\left[{\left(\frac{D_{i}}{d}\right)}^{2}+1\right]}{\left[{\left(\frac{D_{i}}{d}\right)}^{2}-1\right]}+v_{i}\right]+\frac{E_{i}}{E_{a}}\left[\frac{\left[{\left(\frac{d}{d^{\prime }}\right)}^{2}+1\right]}{\left[{\left(\frac{d}{d^{\prime }}\right)}^{2}-1\right]}-v_{a}\right]\right]\left[{\left(\frac{D_{i}}{d}\right)}^{2}-1\right]}\\ \Delta D_{o}=\frac{2\left(I_{o}+\Delta I_{o}\right)\left(\frac{D}{D_{o}}\right)}{\left[\left[\frac{\left[{\left(\frac{D}{D_{o}}\right)}^{2}+1\right]}{\left[{\left(\frac{D}{D_{o}}\right)}^{2}-1\right]}+v_{b}\right]+\frac{E_{o}}{E_{b}}\left[\frac{\left[{\left(\frac{L_{H}}{D}\right)}^{2}+1\right]}{\left[{\left(\frac{L_{H}}{D}\right)}^{2}-1\right]}-v_{o}\right]\right]\left[{\left(\frac{D}{D_{o}}\right)}^{2}-1\right]}\\ \Delta D_{r}=\alpha D_{r}\left(T_{r}-T_{c}\right)\\ \Delta_{r}=\alpha D_{o}\left(T_{o}-T_{c}\right)-\alpha D_{i}\left(T_{i}-T_{c}\right)\\ \Delta I_{i}=\alpha d\left(T_{a}-T_{c}\right)-\alpha d\left(T_{i}-T_{c}\right)\\ \Delta I_{o}=\alpha D\left(T_{o}-T_{c}\right)-\alpha D\left(T_{b}-T_{c}\right) \end{array}\right.$$

where *D_i_* is the inner raceway diameter; *D_o_* is the outer raceway diameter; *D* is the outside diameter; *d* is the bore diameter; *d’* is the shaft inner diameter; *L_H_* is the housing outer diameter; *α* is the linear expansion coefficient; *E* and *ν* are elastic modulus and Poisson ratio; *I* and Δ*I* are interference and increments; *T_c_* is the initial temperature; and *T_r_*, *T_i_*, *T_o_* and *T_b_* are, respectively, the temperatures of the roller, inner ring, outer ring, shaft and bearing housing.

Through the above analysis, the variation law of contact load and velocity of the roller and the relationship between the structural parameters and temperature can be obtained. However, the load distribution form of faulty bearings is obviously different from that of good bearings. Therefore, in order to more accurately calculate the temperature variation law of the faulty bearing during operation, it is necessary to further analyze the load distribution form of the faulty bearing.

### 2.2. The Analysis of Contact Load Distribution of Faulty Bearings

**Figure 2:**
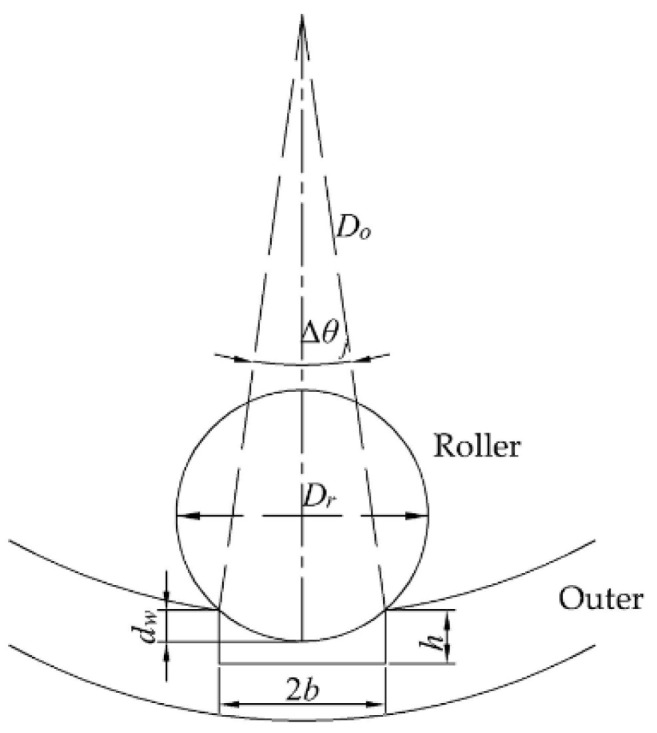
The interaction between the race and the defect.

Take the outer ring as an example. Figure 2 shows the schematic diagram of faulty bearing with a defect on the outer ring. Assuming that the rolling bearing is rigidly supported, the defect depth is h, the defect width is 2b, *θ_j_* is the angular position of the *j*th roller, Δ*θ_j_* is the angle corresponding to the defect width, *θ*_f_ is the angular position of the defect center and *d_ψ_* is the actual depth of the roller into the defect.

Since there is a defect on the outer ring, the contact deformation between the roller and the outer ring should be analyzed in two parts [31]. The contact deformation between roller and good position of the outer ring has been analyzed by a large number of literatures, so it will not be discussed in this paper. The other case is that the roller is in contact with the defect. In the process of calculating load distribution in this situation, it is necessary to estimate whether the *j*th rolling body is in the range of defect, or not. That is, the roller needs to meet the following formula:

(10) $$-\frac{\Delta \theta_{j}}{2}<\mathrm{mod}(\frac{\theta_{j}}{2\pi })-\theta_{f}<\frac{\Delta \theta_{j}}{2}$$

(11) $$\Delta \theta_{j}=2\arcsin\frac{b}{0.5D_{o}}$$

In this case, the contact deformation between the roller and the outer ring is:

(12) $$\delta_{rj}=\delta_{r}\cos\left(\theta_{j}\right)-\frac{1}{2}H_{ol}-d_{\psi }$$

(13) $$d_{\psi }=0.5D_{r}-\sqrt{{\left(0.5D_{r}\right)}^{2}-b^{2}}$$

According to Stribeck’s theory, the relationship between the contact deformation and the maximum contact deformation *δ_r_*_max_ can be obtained:

(14) $$\delta_{rj}=\delta_{r\max}\left[1-\frac{1}{2\varepsilon }\left(1-\cos\left(\theta_{j}\right)\right)\right]$$

where ε is the distribution coefficient, which is related to the bearing clearance *H_ol_* and the total displacement *δ*_r_, and can be obtained according to the method proposed in reference [32].

According to the load-deformation relationship between the rolling body and the outer ring, the relationship among contact load, maximum contact load, contact deformation and maximum contact deformation can be obtained:

(15) $$Q_{rj}=Q_{\max}{\left(\delta_{rj}/\delta_{r\max}\right)}^{10/9}=Q_{\max}{\left[1-\frac{1}{2\varepsilon }\left(1-\cos\left(\theta_{j}\right)\right)\right]}^{10/9}$$

where the maximum contact load can be expressed as:

(16) $$Q_{\max}=K_{rj}{\left(\delta_{r}-0.5H_{ol}\right)}^{10/9}$$

Since the radial load of the bearing is the sum of the loads of each roller, the following formula can be obtained:

(17) $$Q_{r}=Q_{\max}\sum_{\theta_{j}=0}^{\theta_{j}=2\pi }{\left[1-\frac{1}{2\varepsilon }\left(1-\cos\left(\theta_{j}\right)\right)\right]}^{10/9}\cos\left(\theta_{j}\right)$$

According to the above analysis, the load distribution and deformation of each rolling body of the faulty bearing in contact with the inner and outer ring can be obtained, which provides a theoretical basis for the analysis of the heat generation rate of each key component of the bearing.

## 3. Heat Generation and Transfer at Key Positions of Bearing

The heat generated by the bearing mainly comes from the friction between the surfaces of its parts. It includes the friction between the roller and the surface of the inner and outer ring, the friction between the roller and the cage and the friction between the guiding surface of the inner ring and the cage. Compared with the heat generated by other forms of friction, the heat generated by the friction between parts and oil is small and can be negligible, so only the friction heat generated by sliding friction is considered in this section. Normally, the heat generated by bearings is transmitted through heat conduction, heat radiation and heat convection. However, the bearing is installed in a relatively narrow space, so less heat is transferred in the form of thermal radiation, which is not considered in this section.

### 3.1. Heat Generation in Bearing System

(1) Heat generation between roller and raceway. 

The friction heat production between the roller and the inner and outer ring face can be expressed by the following formula:

(18) $$Q_{1}=\mu Q_{ri}\Delta V_{ri}+\mu Q_{ro}\Delta V_{ro}$$

In the above equation, the contact load and relative sliding velocity have been described above, *μ* is the friction coefficient, which is affected by temperature and is related to oil properties, and its calculation formula is [33]:

(19) $$\mu =\left[\frac{4}{\pi p}+\frac{n_{oil}\alpha_{p}}{3}\left(e^{0.707n_{oil}\alpha_{p}p_{0}}+1.866e^{0.259n_{oil}\alpha_{p}p_{0}}+0.134e^{0.966n_{oil}\alpha_{p}p_{0}}\right)\right]\left(\eta_{0}\frac{\Delta V}{h_{c}}\right)G_{r}^{1-n}$$

where *n_oil_* is the power-law exponent of oil, *G_r_* is the shear modulus, *h_c_* is 1.333 times of the minimum oil film thickness, which can be calculated by Equation (8).

(2) Heat generation between roller and cage.

The heat production between the roller and the cage is related to the friction force, and the friction force is limited by the normal force, friction medium, rotation speed and geometry parameters. Therefore, the heat production between a roller and the cage can be expressed by the following formula:

(20) $$Q_{2}=\frac{1}{2}D_{rj}\omega_{rj}F_{rkj}\mu$$

(3) Heat generation between the guide surface of the inner ring and cage.

There is sliding friction between the guide surface of the inner ring and the cage. However, due to the lubricating oil effect between the two parts, the heat generation is not very high, and can be expressed by the following formula:

(21) $$\left\{\begin{array}{l} Q_{k}=\frac{1}{2}d_{m}F_{fk}\omega_{r}\\ F_{fk}=\frac{\eta_{0}\pi w_{k}d_{k}^{2}\omega_{r}}{d_{ik}-d_{k}} \end{array}\right.$$

where *F_fk_* is viscous friction of the oil, *w_k_* is the thickness of the cage and *d_k_* and *d_ik_* are the diameters of the guide surface of the cage and inner ring.

The heat generation of the key component in rolling bearing system can be calculated by the Equations (18), (20) and (21). The total heat generation can be calculated by *N_r_Q*_1_ + *N_r_Q*_2_ + *Q_k_*.

### 3.2. Heat Transfer in Bearing System

**Figure 3:**
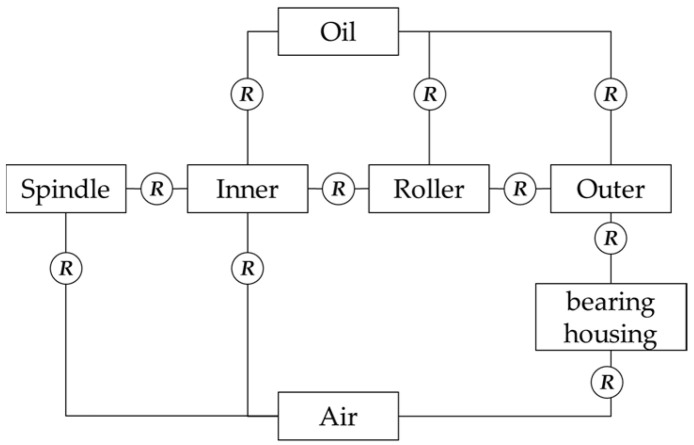
Relation diagram of heat transfer between the parts in bearing.

The heat generated by bearings can usually be transferred by heat conduction and heat convection. Heat is transmitted through the contact between the roller, the inner ring, the outer ring and the cage, and the heat convection mainly occurs between inner ring, outer ring, roller and lubricating oil. The heat transfer relationship between various parts in the bearing is shown in Figure 3, and *R* is the thermal resistance. To determine whether there is heat transfer inside the bearing, it should be judged according to the value of thermal resistance of each bearing component.

#### 3.2.1. The Conduction Thermal Resistance

According to the structural characteristics of bearing parts, the direction of heat conduction can be specifically divided into two kinds: one is conducted along the axial direction, and the other is conducted in the radial direction.

(1) Radial conduction thermal resistance.

For the cylindrical parts such as roller and spindle, the conduction thermal resistance can be calculated by the following formula:

(22) $$\theta =1/\pi K_{D}L_{r}$$

where *K_D_* is the thermal conductivity of the material and *L_r_* is the effective length of the rolling body or shaft.

For the circular ring parts such as outer ring and inner ring, the conduction thermal resistance is:

(23) $$\theta =\ln\left(r_{i}/r_{o}\right)/2\pi K_{D}L_{ring}$$

where *r_i_* and *r_o_* is the diameters of the simplified outer and inner rings, *L_ring_* is the width of the simplified ring.

(2) Axial conduction thermal resistance.

For the calculation of the heat conduction resistance in the axial direction, only the shaft, bearing seat and rollers are considered, and the formula for the calculation is as follows:

(24) $$\theta =L_{cc}/K_{D}A$$

where *L_cc_* is the characteristic length of parts, *A* is the cross-sectional area.

(3) Contact conduction resistance between parts of the rolling bearing.

The most common contact form in bearings is the contact between the roller and the raceway. Based on the Hertz contact theory, the contact conduction resistance of this part can be expressed by the following formula:

(25) $$\theta =\frac{1}{\pi }\left(\frac{a}{b}\right)\frac{1}{k_{D}a\sqrt{V_{c}C_{p}a\rho /k_{D}}}$$

where *a* is the major axis of the contact area, *b* is the minor axis of the contact area, *V_c_* is the characteristic velocity and *C_p_* is the specific heat capacity of the material.

The contact conduction resistance between inner ring and shaft, outer ring and bearing housing can be obtained according to the literature [23].

#### 3.2.2. The Thermal Convection Resistance

In addition to thermal conduction, there are also thermal convection phenomena in the rolling bearing, including free and forced convection. For bearings, this process belongs to forced convection. Convective thermal resistance is related to Nusselt number, and its calculation formula is as follows:

(26) $$\theta_{w}=L_{cc}/AK_{w}Nu$$

where *K_w_* is the thermal conductivity of lubricating oil and *N_u_* is the Nusselt number, which represents the strength of convective heat transfer.

Limited by different operating conditions and thermal convection conditions, the Nusselt numbers are different for different parts, and can be obtained according to the literature [26,33,34].

According to the above analysis of conductive and convective thermal resistance, the corresponding thermal resistance and calculation method of different parts of the faulty bearing system can be obtained by combining Figure 3.

## 4. Node Division and Calculation Process of the Model

### 4.1. Network Note Division

**Figure 4:**
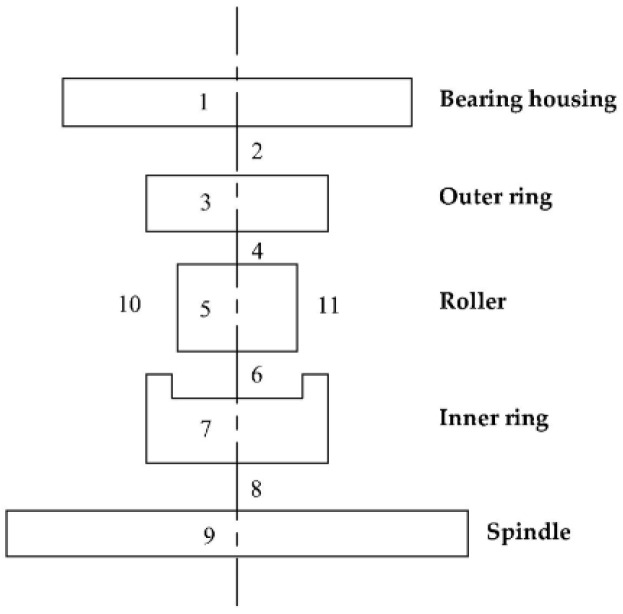
Node division of the rolling bearing system.

Based on the above analysis, according to the structural characteristics of double-row cylindrical roller bearing 130JRF05, the bearing structure is simplified and divided into 11 nodes on its structure, as shown in Figure 4. The node numbers and more details are as follows in Table 1. Due to the limitation of space, the calculation method of the transient temperature of each node can be found by referring to the literature [29].

### 4.2. Transient Temperature Calculation Process of the Faulty Bearing

**Figure 5:**
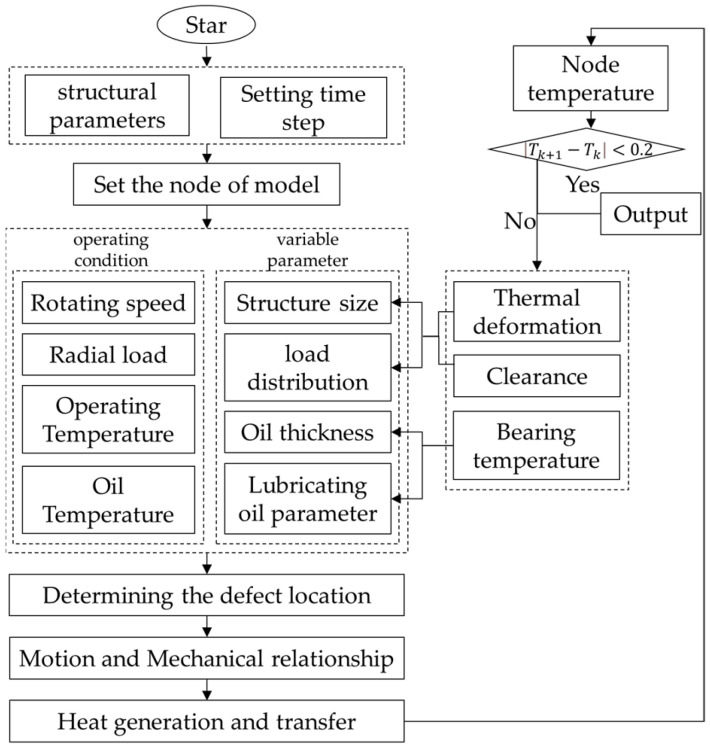
Transient temperature calculation flow of faulty bearing system.

During the operation process of the rolling bearing, the final temperature distribution of the bearing system will be affected by the structure size, running speed, heat production, heat transfer and lubrication effect. In the process of increasing the temperature of the bearing, the thermal system of the bearing is a constantly changing and interacting system. For example, the structural size, working conditions and oil characteristics and other factors will affect the heat generation, and the change of temperature will in turn affect the bearing structure and oil parameters, until the bearing system reaches thermal balance. Therefore, the bearing thermal system is a thermo-solid coupling system. The transient temperature calculation flow of the faulty bearing is shown in Figure 5.

Firstly, the ambient temperature is set as the initial temperature for calculation, and the calculation time interval and bearing structural parameters are set. Secondly, the bearing force is analyzed according to the working conditions, and then the bearing force and deformation under the initial conditions are obtained. Finally, the temperature variation law of bearing is obtained by combining the formula proposed in this paper. In the calculation process, the heat generation and transfer in first step are calculated, and then the obtained calculation results are used as the initial conditions for the next calculation step, and the iterative calculation is carried out continuously. The variable parameters affected by temperature are modified according to the results at the same time.

## 5. Model Validation and Analysis

The proposed model is verified by a rolling bearing temperature measurement experiment platform. The basic parameters of oil are shown in Table 2, and the parameters that change under the influence of temperature are shown in Table 3. The bearings used are cylindrical roller bearings for high-speed trains produced by NSK Company, the model is N205, and its basic structural parameters are shown in Table 4.

**Figure 6:**
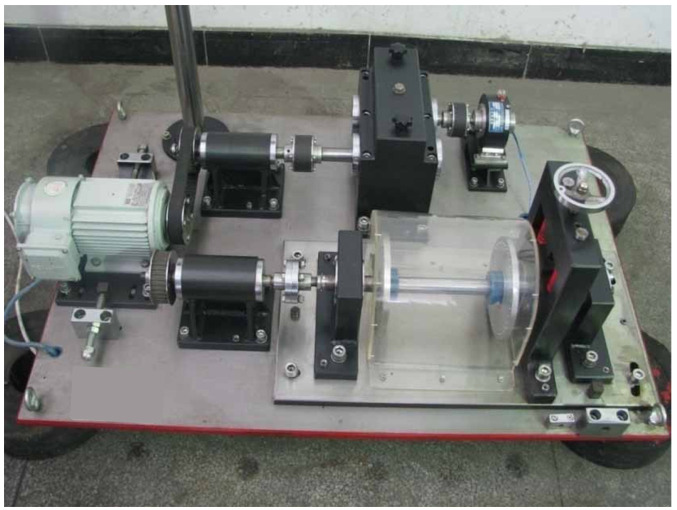
The bearing temperature acquisition and experiment platform.

**Figure 7:**
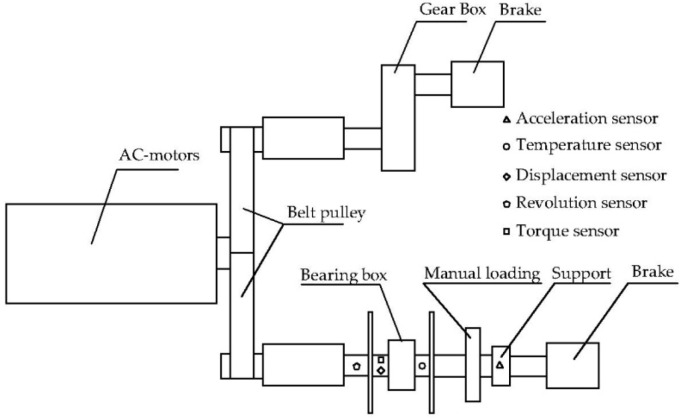
Schematic diagram of experimental platform structure.

The established experimental platform is shown in Figure 6. Through this platform, various states such as rotation and vibration of rotating machinery parts can be quickly simulated, and signals such as temperature, velocity and vibration can be collected. The schematic diagram of the platform structure and the sensor installation position are shown in Figure 7. The experimental platform is composed of an AC variable speed motor, a manual load adjusting device, an electric magnetic powder brake and bearing box, etc. The speed can be adjusted within 0~5000 r/min, and the load can be adjusted within 0~3 Kn. The acquisition card model is NIcDAQ-9178 reconstituted acquisition card, manufactured by National Instruments, Austin, TX, USA. The sampling device includes a dynamic signal acquisition module, a bidirectional digital input module, a thermocouple signal acquisition module and a four-channel synchronous bridge signal acquisition module. During the experiment, the temperature is collected by digital channel. The components of the signal acquisition system are connected with each other by sensor signal lines, and there is no crossover phenomenon between lines. The defect of the faulty bearing used in the experiment is located in the outer ring, with a depth of 0.5 mm and a width of 3 mm. In the calculation, the initial temperature is set as the temperature of the experimental environment. In the experimental process, the temperature rise process and thermal equilibrium temperature of good and damaged bearings were compared under the same load and different speeds and under the same speed and different loads.

### 5.1. The Influence of the Rotational Velocity on Temperature

**Figure 8:**
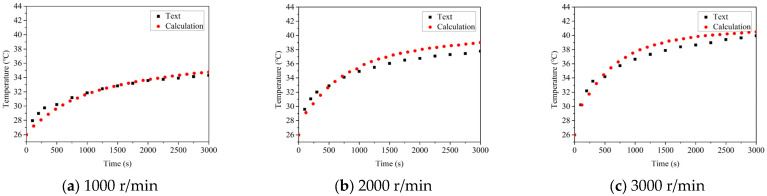
The influence of the speed on temperature change of complete bearing.

**Figure 9:**
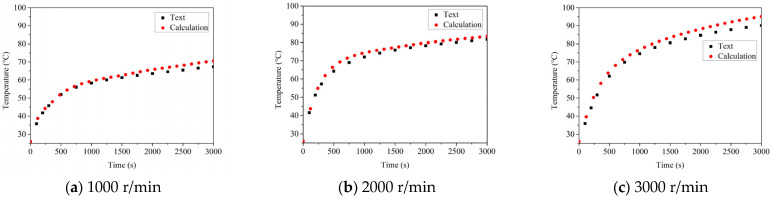
The influence of the speed on temperature change of damaged bearing.

The temperature rises and thermal balance temperature of the good bearing and the defective bearing at the speed of 1000 r/min, 2000 r/min and 3000 r/min are compared, as shown in Figure 8 and Figure 9, respectively. It can be found that the temperature rise process of good bearings is similar regardless of the speed of the bearings. Before the 2000 s, the temperature increased slowly and reached a stable state before 3000 s. However, the thermal balance temperature of the bearing will increase with the increase of the rotation speed. According to the previous analysis, the influence of speed on bearing temperature is very significant, and the increase of speed also leads to the increase of heat generation between parts of the rolling bearing, which leads to the higher temperature of bearing to reach thermal balance. As can be seen from the figure, when the speed reaches 3000 r/min, the bearing temperature increases by about 6 °C compared with that at 1000 r/min, which can also be observed from the experimental results.

Reviewing the temperature rise process of the damaged bearing, it can be found that the temperature is also rising in 1000 s of the bearing operation, but the climbing rate is obviously higher than that of the good bearing. After 1000 s, the temperature still rises, but the rate of temperature rise decreases significantly, which is because the bearing structure is close to the limit of thermal expansion, leading to the gradual delay of heat production. At the same time, by comparing the thermal balance temperature of the two kinds of bearings at different speeds, it can be found that the thermal balance temperature of the faulty bearing is significantly higher than that of the good bearing. It is about 3~4 times that of the thermal equilibrium temperature of the good bearing, and the highest temperature reaches 95.06 °C, which is in good agreement with the experimental results. Through the error analysis of the results, the errors of good bearings at different speeds are 4.72%, 7.76% and 8.54%, respectively. The errors of faulty bearings at different speeds are 5.35%, 6.81% and 7.93%, respectively. The error is within the allowable range, which proves the correctness of the model.

### 5.2. The Influence of the Load on Temperature

**Figure 10:**
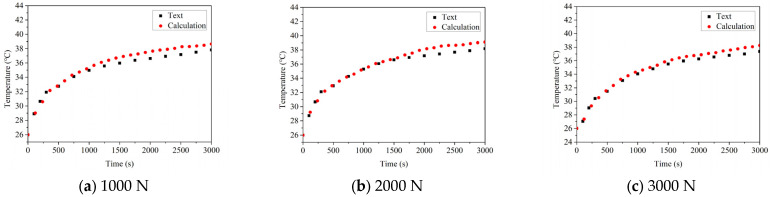
The influence of the load on temperature change of complete bearing.

**Figure 11:**
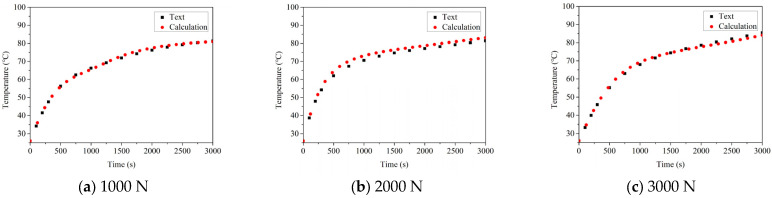
The influence of the load on temperature change of damaged bearing.

The temperature rises and thermal balance temperature of the good bearing and the defective bearing at the speed of 1000 N, 2000 N and 3000 N are compared, as shown in Figure 10 and Figure 11, respectively. In the case of the good bearing, the temperature increases slowly with the running time of bearing, and finally reaches the thermal equilibrium state. Before reaching the stable state, the temperature rise rate does not change significantly. At the same time, it can be found that the temperature of the good bearing thermal equilibrium under different loads shows an upward trend, but the amplitude of temperature increase is small, which indicates that the radial load has no obvious influence on the temperature of the good bearing.

However, another phenomenon can be found by observing the temperature rise of the faulty bearing. In the initial operation stage of the bearing, the temperature rises sharply, and the temperature rise rate can clearly see the boundary position. When the bearing structure is close to the limit size of thermal expansion, the temperature rise rate decreases. It can also be found that with the increase of load, the maximum temperature of the bearing gradually increases, and the amplitude of the lifting is significantly higher than that of the good bearing. However, compared with the influence of bearing speed on temperature of thermal equilibrium state, the influence of load is not very obvious. This should be attributed to the fact that when the bearing has defects, with the increase of the radial load of the bearing, the contact load between the roller and the defect position will also be increased, which will result in the increase of heat generation between the roller and the outer ring, and then the temperature of the bearing will be increased. The same phenomenon can be also observed in the data obtained from the experiment. Based on the error analysis of the calculation results and experimental results, it can be found that the errors are 4.63%, 3.47% and 3.36%, respectively, under the condition of bearing without damage, and 2.87%, 3.12% and 2.66%, respectively, under the condition of bearing with defects. The error is also within the allowable range.

According to the experimental results and analysis above, the correctness of the established model is proved. The established bearing thermal analysis model, which, considering the influence of thermal-solid coupling, can effectively obtain the temperature variation law and thermal equilibrium temperature of damaged bearings under different operating conditions. It can also be found that when there is a fault in the bearing, the bearing speed has a more obvious influence on the bearing temperature compared with the radial load.

## 6. Conclusions

In this paper, a novel thermal analysis model of faulty bearing system is established, which considers the effects of thermal-solid coupling on the temperature variation. The influence of rotation speed and radial load on temperature variation of faulty bearing system is analyzed. The correctness of the established model is verified by experiments, and the conclusions are as follows:

(1) The coupling relationship between bearing heat generation and bearing structure, lubrication oil and working conditions was studied, and the transient temperature calculation model of faulty bearing was established with the method of thermal network. The heat generation rate and conduction resistance between friction surfaces of bearing were calculated, and the influence of thermo-solid coupling on temperature variation and thermal equilibrium temperature was obtained.

(2) Under the same working condition, the rate of heat generation and the thermal equilibrium temperature of the defective bearing are higher than those of the good bearing, because the defect changes the distribution of the contact load of the roller.

(3) Compared with the radial load, the rotate speed has a greater impact on the heat generation and thermal equilibrium temperature of the bearing, which is more obvious in the faulty rolling bearing.

The proposed model can effectively obtain the temperature of faulty bearings. The influence of temperature on vibration signal of faulty bearing can be further analyzed and applied to fault detection of bearing, such as acoustic detection, temperature detection and so on. However, this model still has some limitations: the lubricating oil of the bearing is constantly running and takes heat away, which is not considered in this paper. Therefore, the influence of flowing lubricating oil on the temperature of faulty bearings will be considered in the next study.

## Figures and Tables

**Table 1:** The description of nodes.

Nobel Number	Description	Nobel Number	Description
1	Bearing housing	7	Inner ring
2	Contact of bearing housing and outer ring	8	Contact of inner ring and spindle
3	Outer ring	9	Spindle
4	Contact of outer ring and roller	10	Oil in bearing box
5	Roller	11	Air
6	Contact of roller and inner ring		

**Table 2:** Basic parameters of the lubricating oil.

Parameters	Value
exponent sign (*n_oil_*)	0.43
shear modulus (*G_r_*, Pa)	$5.31\times 10^{4}$
thermal conductivity (*K_w_*, W/m °C^−^^1^)	0.132
density (*ρ*, kg/m^3^)	920

**Table 3:** Lubricating oil parameters after affected by temperature.

Temperature (°C)	Kinematic Viscosity $\left({\mathrm{Ns}/m}^{2}\right)$	Pressure–Viscosity Coefficient $\left(m^{2}/N\right)$	Dynamic Viscosity $\left(m^{2}/s\right)$
0	$6.10\times 10^{-1}$	$3.03\times 10^{-8}$	$5.10\times 10^{-1}$
10	$3.12\times 10^{-1}$	$2.65\times 10^{-8}$	$3.05\times 10^{-1}$
20	$2.05\times 10^{-1}$	$2.13\times 10^{-8}$	$1.85\times 10^{-1}$
30	$1.31\times 10^{-1}$	$1.83\times 10^{-8}$	$1.23\times 10^{-1}$
40	$0.93\times 10^{-1}$	$1.75\times 10^{-8}$	$0.81\times 10^{-1}$
50	$0.47\times 10^{-1}$	$1.63\times 10^{-8}$	$0.32\times 10^{-1}$
60	$0.33\times 10^{-1}$	$1.57\times 10^{-8}$	$0.21\times 10^{-1}$
70	$0.19\times 10^{-1}$	$1.45\times 10^{-8}$	$0.12\times 10^{-1}$
80	$0.12\times 10^{-1}$	$1.32\times 10^{-8}$	$0.08\times 10^{-1}$
90	$0.08\times 10^{-1}$	$1.22\times 10^{-8}$	$0.04\times 10^{-1}$

**Table 4:** Basic parameters of the N205.

Parameters	Value
Outerdiameter (mm)	52
Inner diameter (mm)	25
Width (mm)	15
Roller diameter (mm)	7.02
Pitch diameter (mm)	38.5
Number of Rollers	13
Bearing clearance (mm)	0.1
Interference (mm)	0.01

## Data Availability

All data used to support the findings of this study are included within the article.
